# The Janus-like role of proline metabolism in cancer

**DOI:** 10.1038/s41420-020-00341-8

**Published:** 2020-10-14

**Authors:** Lynsey Burke, Inna Guterman, Raquel Palacios Gallego, Robert G. Britton, Daniel Burschowsky, Cristina Tufarelli, Alessandro Rufini

**Affiliations:** 1 Leicester Cancer Research Centre, University of Leicester, Leicester, LE2 7LX UK; 2 Leicester Institute of Structural and Chemical Biology and the Department of Molecular and Cell Biology, University of Leicester, Lancaster Road, Leicester, LE1 7HB UK

**Keywords:** Cancer metabolism, Cancer

## Abstract

The metabolism of the non-essential amino acid L-proline is emerging as a key pathway in the metabolic rewiring that sustains cancer cells proliferation, survival and metastatic spread. Pyrroline-5-carboxylate reductase (PYCR) and proline dehydrogenase (PRODH) enzymes, which catalyze the last step in proline biosynthesis and the first step of its catabolism, respectively, have been extensively associated with the progression of several malignancies, and have been exposed as potential targets for anticancer drug development. As investigations into the links between proline metabolism and cancer accumulate, the complexity, and sometimes contradictory nature of this interaction emerge. It is clear that the role of proline metabolism enzymes in cancer depends on tumor type, with different cancers and cancer-related phenotypes displaying different dependencies on these enzymes. Unexpectedly, the outcome of rewiring proline metabolism also differs between conditions of nutrient and oxygen limitation. Here, we provide a comprehensive review of proline metabolism in cancer; we collate the experimental evidence that links proline metabolism with the different aspects of cancer progression and critically discuss the potential mechanisms involved.

## Facts

- Proline metabolism is widely rewired during cancer development.
- The rewiring of proline metabolism influences numerous physiological pathways, including mitochondrial metabolism, apoptosis, protein synthesis.
- PRODH acts as a tumor suppressor or an oncogene depending on the tumor type and the environmental, metabolic context.
- PYCR1 acts as an oncogene and is overexpressed in a wide variety of malignancies.

## Open questions

- What is the impact of the interplay between proline biosynthesis and degradation in cancer?
- How is the rewiring of proline metabolism regulated depending on cancer type and cancer subtype?
- Is it possible to develop successful pharmacological inhibitor of proline metabolism enzymes for anticancer therapy? And to what extent would these inhibitors be useful in conjunction with standard of care chemotherapies?
- Does the rewiring of proline metabolism affect the anti-tumor immune response and cancer immune evasion?
- To what extent and with what consequences are proline metabolism enzymes post-translational modified?

## Introduction

In their revised version of the hallmarks of cancer, Hanahan and Weinberg introduced the new hallmark ‘deregulating cellular energetics’^1^ in acknowledgement of the growing amount of literature that flourished around the rediscovery of Otto Warburg’s observation of deregulated glucose metabolism in cancer cells. During the 1920s, Warburg demonstrated that cultured tumor tissues display high rates of glucose uptake and lactate secretion, even in the presence of oxygen, a phenomenon known as aerobic glycolysis or the Warburg effect^2,3^. In the wake of the revival of Warburg’s work, numerous discoveries led to the appreciation that cancer cells undergo extensive metabolic reprogramming to preserve redox and energetic balance while meeting the metabolic demand imposed by rapid proliferation^4,5^. Depending on the cancer type and its tissue of origin, alterations in diverse metabolic pathways have been identified that are involved in the metabolism of the main biological macromolecules: lipids, nucleic acids, carbohydrates, and amino acids, including non-essential amino acids (NEAAs)^6^.

The rewiring of NEAA metabolism bears clinically relevant consequences^7^. L-asparaginase has been used to treat acute lymphoblastic leukemia and non-Hodgkin lymphomas since the 1970s. Asparaginase enzymes deaminate L-asparagine to aspartic acid and ammonia. Since leukemia cells require external supplementation of the NEAA L-asparagine, its depletion by asparaginase treatment is lethal to tumor cells. The efficacy and selectivity of this treatment result from cancer cells downregulating the expression of the asparagine synthetase *ASNS* gene^8^. Similarly, some cancers silence the expression of the argininosuccinate synthase-1 *ASS1* gene, which encodes the enzyme that catalyzes the condensation of citrulline and aspartate to form argininosuccinate in the urea cycle^9^. *ASS1* silencing sustains proliferation by diverting aspartate away from the urea cycle toward nucleotide biosynthesis^10^, but, concomitantly, results in cancer cells becoming auxotrophic (i.e., depending on external supplementation) for arginine. Preclinical studies and clinical trials have confirmed that ASS1-negative cancers are susceptible to arginine deprivation therapies using mycoplasma-derived arginine deiminase or recombinant human arginase^11–26^.

In recent years, adaptations of other NEAA metabolic pathways have been associated with cancer progression^7,27^. Enthusiasm and research efforts have grown with regard to the possibility of targeting those pathways for developing new cancer therapeutics^7^. This review focuses on the NEAA proline and the experimental evidence that associates its unique metabolism to cancer.

## The essential NEAA L-proline

**Fig. 1:**
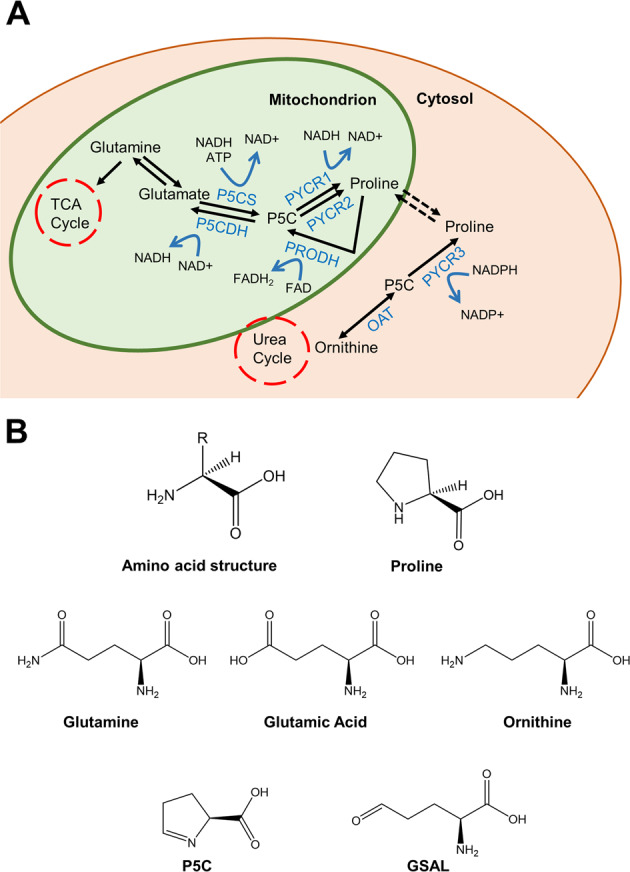
The proline metabolic pathway. **A** The proline metabolic pathway. The NEAA L-proline is formed through reduction of precursor P5C that is obtained via two pathways, from glutamine in the mitochondria and from ornithine in the cytosol. **B** Chemical structures of the proline metabolic pathway intermediates. Note the secondary amine group in proline is different from that of other amino acids. Produced in Chemdraw.

L-proline has a distinctive structure compared to other proteinogenic amino acids, as it lacks the primary amine group and instead has a secondary amine due to the nitrogen group covalently binding the alpha carbon to form a five-membered imino ring (Fig. 1). This unique conformation grants L-proline essential properties in influencing the 3D structure of proteins^28^. L-proline is known as a ‘helix breaker’ due to its ability to disrupt the α-helix conformation by introducing a kink. Proline kinks play important roles in influencing the 3D structure of proteins, including transmembrane helices^29,30^. In addition, proline-rich motifs within proteins mediate critical protein–protein interactions^31^.

L-proline can also be modified for regulatory purposes. For example, *cis*–*trans*-isomerization of L-proline residues by peptidyl-prolyl isomerases controls protein folding and function, with important implications for tumorigenesis^32,33^. L-proline hydroxylation is a well know post-translational modification of proteins. Hydroxylation of L-proline can have critical regulatory functions, as in the case of the transcription factor hypoxia-inducible factor (HIF) 1, which mediates cellular response to oxygen deprivation. HIF1 is a dimer of a constitutively expressed HIF1-β subunit and an oxygen-sensitive HIF1-α subunit^34^ that accumulates in hypoxic cells. HIF1-α protein contains two proline residues (P402/P564) that are hydroxylated in the presence of oxygen by prolyl hydroxylase domain-containing proteins (PHDs). Hydroxylation of the proline residues is a pre-requisite for von Hippel–Lindau-mediated ubiquitination and degradation of HIF1-α, preventing its accumulation in oxygenated tissues^34^.

L-proline is also the most abundant amino acid contributing to the composition of the extracellular matrix (ECM). Collagen, the main structural protein of the ECM, contains ~25% of L-proline residues. When L-proline is incorporated into collagen, its hydroxylation by prolyl hydroxylases generates 4-hydroxyproline. This high L-proline content stabilizes collagen molecules with 4-hydroxyproline having the biggest stabilizing effect. The positioning of these residues is also important for recognition and subsequent degradation of collagen by matrix metalloproteinases^35–37^. Moreover, intracellular hydroxyproline can originate from collagen digestion by prolidase, a cytosolic dipeptidase that breaks down dipeptides to produce free L-proline and L-hydroxyproline^36^.

## Proline metabolism

The metabolic pathways that control proline biosynthesis and catabolism are distinct from that of other NEAAs, as the common pyridoxal-phosphate-coupled transaminases cannot metabolize the L-proline secondary amine.

The biosynthesis of L-proline occurs from glutamine in the mitochondria and ornithine in the cytosol with both precursors converging to the intermediate glutamate-γ-semialdehyde (GSAL), which exists in spontaneous equilibrium with the ring-structured pyrroline-5-carboxylate (P5C) (Fig. 1). In the cytosol, ornithine is converted to GSAL/P5C by the action of the pyridoxal-phosphate dependent, mitochondrial enzyme ornithine aminotransferase (OAT), which is also responsible for catalyzing the reverse reaction. In the mitochondria, glutamate is converted to GSAL/P5C by the mitochondrial enzyme 1-pyrroline-5-carboxylate synthase (P5CS, encoded by the *ALDH18A1* gene, chromosome 10q24.1). In eukaryotes, P5CS is a bifunctional enzyme, which contains an N-terminal glutamate kinase domain and a C-terminal γ-glutamyl phosphate reductase (GPR) domain. P5CS phosphorylates and then reduces glutamate to P5C, and requires both ATP and NADH as cofactors^38^. The P5CS protein has two alternative spliced forms. The shorter isoform is highly expressed in the gut, whereas the longer isoform, which differs from the shorter by the addition of two amino acids in the GPR domain, is ubiquitous^39^.

In the final reaction, P5C is converted to L-proline by the activity of NAD(P)H-dependent PYCR enzymes. There are three homologous PYCR isoforms: PYCR1, PYCR2, PYCR3 (aka PYCRL), which are encoded for by separate genes, namely PYCR1 (chromosome 17q25.3), PYCR2 (chromosome 1q42.12), and PYCR3 (chromosome 8q24.3). In addition, each isoform is encoded by several poorly characterized splice variants (Table 1). PYCR3 is the only proline metabolism enzyme to be localized in the cytoplasm, and its similarity to the other PYCR isoforms is around 45%. PYCR1 and 2 share 85% sequence similarity and have been reported to heterodimerize and to be localized to the mitochondria^40–42^. However, the exact localization of PYCR1 and 2 within the mitochondrion is not known, and PYCR1 has been suggested to localize to the outer mitochondrial membrane and, partially, to the cytoplasm^42,43^. PYCR isoforms differ in their cofactor and substrate affinities. Current data indicate that PYCR1 and 2 have a higher affinity for the cofactor NADH, whereas the rate of conversion of P5C by PYCR3 is higher in the presence of NADPH^41,44^. Moreover, isotope enrichment experiments in melanoma cell lines showed that PYCR1 and PYCR2 primarily catalyze the synthesis of L-proline from glutamate, although, in the presence of high levels of ornithine, PYCR1 can also produce L-proline through ornithine. The same experiments also indicated that PYCR3 works exclusively along the ornithine route^41^.

**Table 1:** Splice variants of the three PYCR isoforms as identified on NCBI.

Isoform	Gene location	NCBI reference	Transcript variant	mRNA length	Amino acid length
PYCR1	17q25.3	NP_008838.2	1	2269	319
		NP_722546.1	2	1926	316
		NP_001269208.1	3	1776	288
		NP_001269209.1	4	1869	319
		NP_001269210.1	5	1951	346
		NP_001317452.1	6	1705	217
PYCR2	1q42.12	NP_037460.2	1	1680	320
		NP_001258610.1	2	1458	246
PYCR3	8q24.3	NP_075566.3	1	3342	274
		NP_001316795.2	2	3282	254

PYCR1 transcript variants 1 and 4 code for the same protein.

**Fig. 2:**
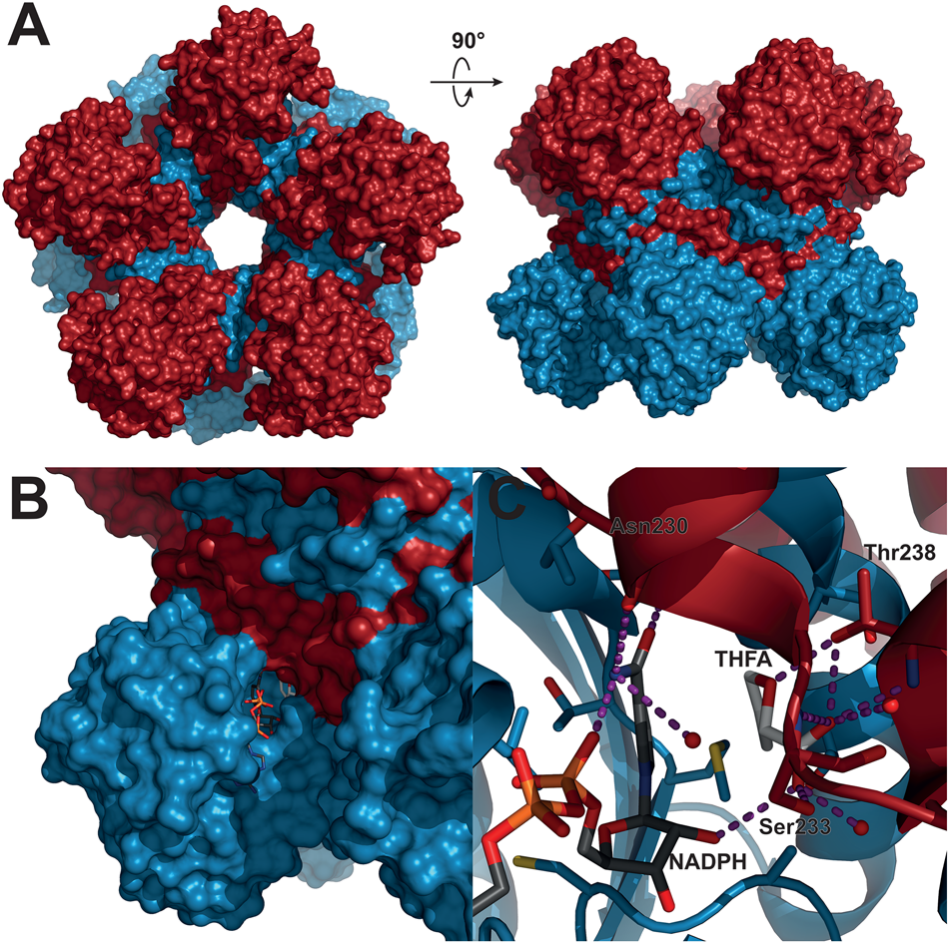
Detailed 3D structure of the human PYCR1 enzyme. **A** Overview of space-filling model of PYCR1 (PDB ID 5UAV, ref. ^44^). PYCR1 forms a pentamer of dimers, with individual dimer subunits colored blue and red, respectively. **B** Zoomed view of the NADPH binding site and the active site. NADPH is bound to the N-terminal Rossmann-fold dinucleotide-binding domain, which forms the outer part of the protomer, whereas the P5C/proline analog L-tetrahydrofuroic acid (THFA) interacts with the C-terminal dimerization domains of both protomers. NADPH and THFA are shown as sticks with dark gray and light gray carbons, respectively. **C** Close-up cartoon of the active site of PYCR1. Residues within 4 Å of THFA or the nicotinamide moiety of NADPH are shown as sticks, water oxygens are shown as red spheres, and hydrogen bonds are highlighted with purple dashes. The substrate analog is mainly stabilized by a hydrogen bond network with the backbone and side-chain atoms of Ser233 and Thr238 and 2 water molecules. The images were prepared using PyMOL 2.3.5 (ref. ^45^).

A detailed 3D structure of the human PYCR1 protein has been solved by Christensen and colleagues (Fig. 2)^44,45^. This work confirmed previous observations that in vivo monomeric PYCR1 proteins coalesce into a decameric structure^46^ or, more accurately, a pentamer of dimers of over 350 kDa. Each monomeric enzyme consists of an N-terminal Rossmann-fold domain for binding of NAD(P)H and a six α-helix C-terminal dimerization domain. The substrate P5C binds at the interface between two dimers with its ring positioned in parallel to nicotinamide, such that the hydride acceptor atom of P5C lies in proximity to the C4 carbon donor of nicotinamide, suggesting a direct hydride-transfer mechanism for the reduction of P5C^44^.

The catabolism of L-proline follows an opposite pathway to its biosynthesis catalyzed by different enzymes. The first step is mediated by the PRODH (aka proline oxidase, POX) enzymes, which catalyze the FADH-dependent oxidation of proline to P5C. There are two PRODH enzymes; PRODH1, the primary enzyme in the conversion of L-proline back to P5C, coded by the *PRODH1* gene (chromosome 22q.11.21), and PRODH2, coded by the *PRODH2* gene (chromosome 19q13.12), which catalyzes hydroxyproline conversion to pyrroline-3-hydroxy-5-carboxylate^47^. PRODH1 is conserved throughout evolution although, in some prokaryotes, it is fused with pyrroline-5-carboxylate dehydrogenase (P5CDH) to form the Proline Utilization A (PutA) flavoprotein. A study of the *Escherichia coli* homolog of the PRODH portion of PutA showed a dimeric structure with each subunit containing a dimerization domain, a three-helix bundle, and a β/α-barrel catalytic PRODH domain^48,49^. The residues that are involved in proline binding are conserved across different organisms, suggesting that structural properties of the prokaryotic enzymes likely apply to their eukaryotic counterpart^50^. L-proline catabolism is coupled to mitochondrial respiration through the FADH-mediated transfer of electrons to the electron transport chain (ETC) or direct interaction between PRODH and coenzyme Q^51–53^.

PRODH activity generates GSAL/P5C. GSAL/P5C can be metabolized to ornithine by OAT, linking proline metabolism to the urea cycle^54^. Alternatively, GSAL is oxidized by NAD-dependent mitochondrial enzyme P5CDH (*ALDH4A1*, chromosome 1p36.13) to yield glutamate^55–57^. P5CDH displays a high degree of conservation across prokaryotes and eukaryotes and has a typical ALDH structure, consisting of a NAD^+^-binding non-canonical Rossmann fold, a catalytic β/α-barrel domain, and a β structured oligomerization domain^58^. Two subunits interact via the β sheets of the catalytic domain to form a dimeric structure^50^. In *Thermus thermophiles*, bacterial P5CDH was shown to form a hexameric complex with two other dimers^59^.

From this summary, it emerges that the metabolism of proline is wired with other key metabolic pathways, including the urea cycle through ornithine and the tricarboxylic acid (TCA) cycle through glutamate, which can be promptly metabolized to the TCA cycle intermediate α-ketoglutarate by glutamate dehydrogenase or through transamination. In addition, through redox regulation of cofactors NADH and FADH, proline metabolism influences the activity of mitochondrial TCA and ETC^51,52^. Although it remains to be fully elucidated, the ability of PYCR3 and, possibly, PYCR1 to metabolize NADPH within the cytoplasm might stimulate the pentose phosphate pathway (PPP) with important implication for ribose biosynthesis and nucleotide metabolism^60^. It is noteworthy that, other than de novo biosynthesis or nutrient uptake, breakdown of collagen is an important reservoir of L-proline, which, as outlined below, can be exploited by cancer cells^43,61,62^.

## Regulation of the proline metabolic pathway

**Fig. 3:**
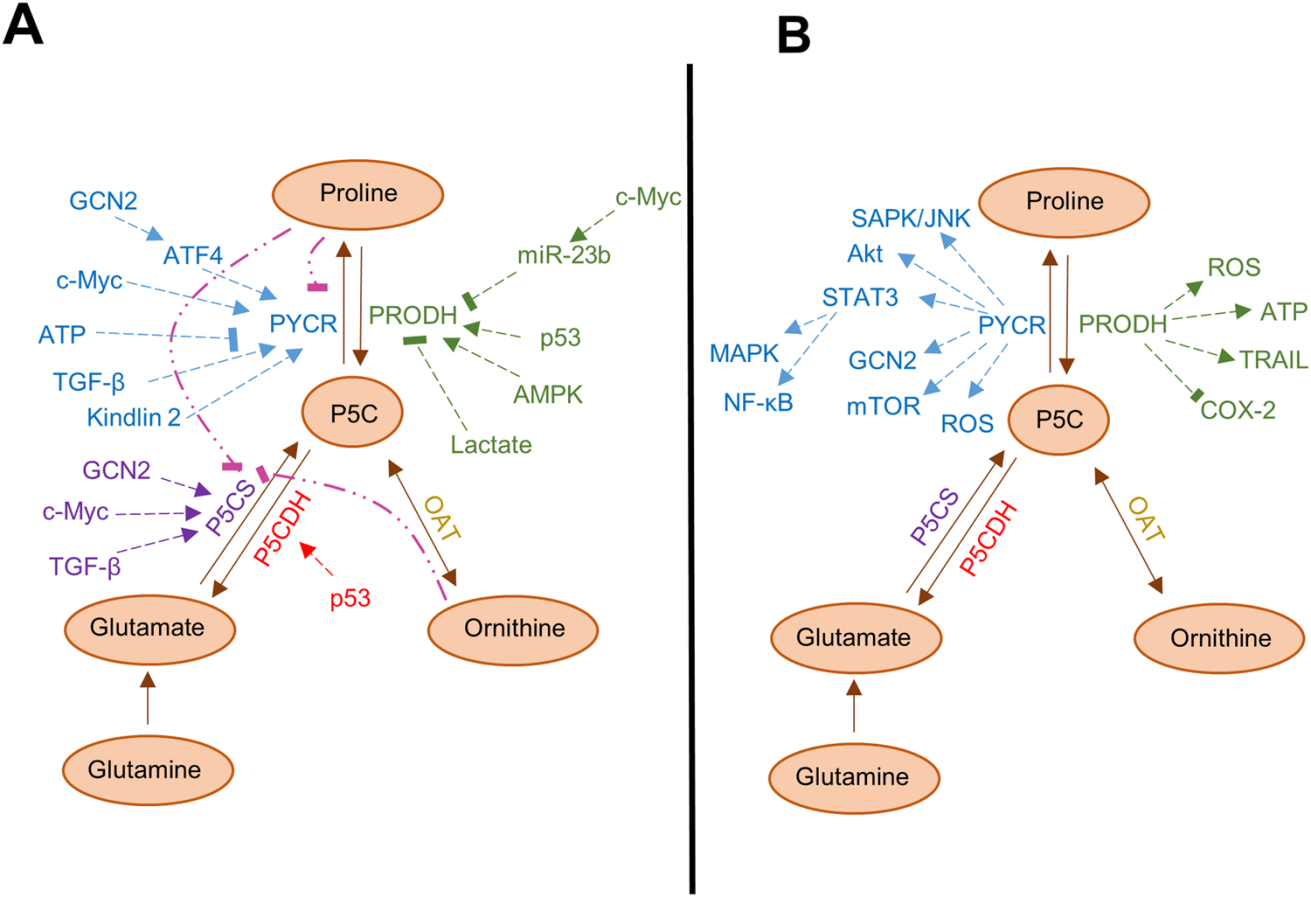
Proline metabolism is regulated by many cellular pathways and influence key cellular signaling nodes. **A** Regulation of the proline metabolic pathway is supported by the activity of numerous signaling pathways (→). Feedback inhibition loops (**--|**) also contribute to regulate the rate of the proline metabolism pathway. **B** The proline metabolic pathway enzymes regulate signaling activity of numerous pathways both positively (→) and negatively (**--|**).

When L-proline levels are high, negative feedback loops are activated in order to decrease biosynthesis. Both P5CS and PYCR1 are inhibited by the final product of the pathway, L-proline, though the mechanism of this inhibition is still elusive (Fig. 3). In vitro data indicate that, of the three PYCR isoforms, PYCR2 is the most sensitive to proline inhibition, showing a robust decline in enzymatic activity at physiological concentrations of L-proline (0.1–0.3 mM)^41^, whereas PYCR1 and PYCR3 showed limited product feedback inhibition^41^. It will be important to ascertain the implications of these dissimilar behaviors for proline metabolism rate in vivo. Moreover, PYCR enzymes have been reported to be sensitive to inhibition by ATP^63^. On the other hand, studies in renal cell carcinoma cell lines showed that reducing the level of glutamine in cell culture causes increased transcription of the *PYCR1* gene, but not its paralogues^64^. Glutamine can be deaminated to glutamate by glutaminase enzymes and, therefore, it is a major precursor of proline biosynthesis. Hence, increased expression of biosynthesis enzymes compensates for reduced availability of L-proline precursors.

Ornithine can inhibit P5CS activity in an isoform-dependent fashion^38,39^. Whereas the short P5CS isoform is inhibited non-competitively by ornithine, the two amino acid insertion that mark the long isoform abolishes the feedback inhibition by ornithine. It is thought that ornithine inhibition rewires proline metabolism to favor arginine biosynthesis^38,39^. Indeed, high ornithine levels have been shown to inhibit the biosynthesis of L-proline from glutamate through reduced activity of P5CS^38,39^. Since the long isoform of P5CS is ubiquitous, whereas the short one is preferentially expressed in the gut, a channeling of proline metabolism toward arginine biosynthesis likely occurs in the gut, whereas other tissues primarily synthesize L-proline^38,65^.

PRODH has been shown to be inhibited by lactate^66^. Lactate is the product of anaerobic respiration and is produced by cancer cells that display enhanced aerobic glycolysis. Therefore, the inhibition of PRODH by lactate indicates PRODH activity might be negatively affected in cancer cells.

## The ambivalent role of *PRODH* in cancer

Currently, no mutations have been described in the proline metabolism genes that have been functionally linked to tumor development. However, changes in gene expression and metabolic flux through the pathway have been extensively reported. The majority of the experimental evidence that associates the metabolism of L-proline to cancer development revolves around the two genes that catalyze the last step in L-proline biosynthesis and the first step of its catabolism, namely *PYCR1* and *PRODH1*. A simplified view of those genes’ modus operandi in cancer would implicate proline biosynthesis by the PYCR enzymes in cancer progression and its catabolism by PRODH in the suppression of tumorigenesis. However, the regulation and outcome of these enzymes’ activities in cancer progression are mostly context-dependent and exceedingly more complex (Fig. 3).

**Fig. 4:**
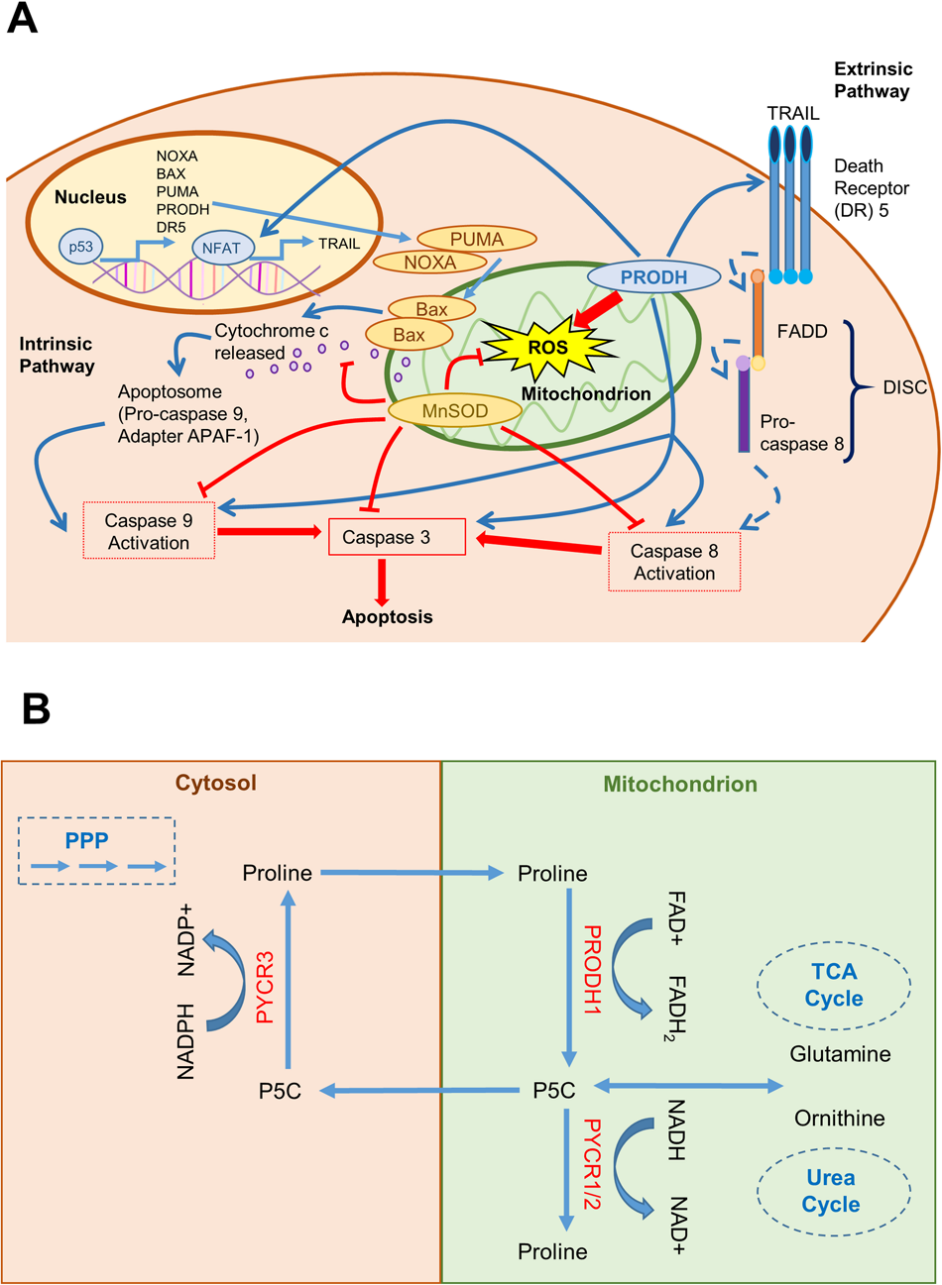
Proline metabolism in the regulation of cell survial and redox balance. **A** PRODH has been implicated in the activation of apoptosis via both the intrinsic and extrinsic pathways. Activation of the intrinsic pathway by reactive oxygen species (ROS) is mediated by the p53-induced transcriptional activation of genes, including PRODH1. In the intrinsic pathway, the BH3-only proteins, such as NOXA and PUMA, inactivate the pro-survival proteins such as BCL-2 and lead to the release of pro-apoptotic proteins such as BAX. This allows the permeabilization of the mitochondrial outer membrane. Cytochrome c is released from the mitochondria and stimulates assembly of the apoptosome containing APAF-1 and pro-caspase 9. The apoptosome leads to the activation of caspase 9, which, in turn, activates the effector caspase 3 resulting in apoptosis. The extrinsic pathway of apoptosis is initiated by the activation of the death receptor of the TNF superfamily, such FAS and TRAIL. Receptor engagement and trimerization lead to the recruitment and activation of caspase-8 in the death inducing signaling complex (DISC), which in turn activates the effector caspase 3, resulting in apoptosis. PRODH1 has been shown to induce expression of the death receptor 5 (DR5) and its ligand TRAIL through stimulation of the nuclear factor of activated T cells (NFAT) transcription factor. **B** Schematic representation of the proposed proline cycle. L-proline is oxidized to P5C by PRODH1 in the mitochondria. P5C is then reduced back to L-proline through the action of the PYCR3 enzyme in the cytosol, a reaction coupled with the oxidation of NADPH. The cycle shuttles electrons from the cytosol to the mitochondria and stimulates flux through the PPP via oxidation of NADPH. In this way, the proline cycle can potentially contribute to nucleotide biosynthesis.

*PRODH1* was identified in 1997 as *PIG6*, a pro-oxidative gene upregulated in response to the ectopic expression of the tumor suppressor p53 in colorectal cancer cell lines^67^. *PRODH* was thus implicated in the ROS-dependent apoptotic response to p53 induction^68^. Notably, a follow-up work indicated that overexpression of *PRODH1* was sufficient to induce apoptosis in a p53-resistant bladder cancer cell line^68^. Subsequent reports confirmed the ability of *PRODH1* and, to some extent *PRODH2*, to induce ROS, in particular anion superoxide, trigger apoptosis in a ROS-dependent fashion and act as a tumor suppressor^69–72^. Intriguingly, ectopic expression of PRODH1 in DLD-1 colorectal cancer cell lines triggers apoptosis through multiple mechanisms (Fig. 4). In addition to ROS-dependent intrinsic apoptosis^73^, PRODH induces expression of the death receptor TRAIL, and inhibition of caspase-8 activity is effective in restricting apoptosis in PRODH1-overexpressing cells, implicating activation of the extrinsic apoptotic pathway^74,75^. Finally, these apoptotic responses are enabled by a robust ROS-dependent downregulation of the ERK1/2 branch of the mitogen-activated protein kinase (MAPK) pathway, which has been widely implicated in malignant progression^74^. Indeed, expression of the ROS scavenging enzyme manganese superoxide dismutase (MnSOD) inhibits PRODH1-induced apoptosis^74^. However, the contribution of PRODH1-induced apoptosis to tumor suppression in vivo is unclear. Indeed, xenograft experiments performed using DLD-1 colorectal cancer cells engineered to overexpress PRODH1 showed that the reduction in tumor growth triggered by PRODH1 expression correlated only with marginal induction of cell death, but with markedly reduced proliferation^71^. A follow-up in vitro experiment led to the appreciation that the phenotypic outcome of PRODH1 expression depends on quantity: high levels of PRODH1 cause apoptosis, whereas lower levels of expression prompt a cell cycle arrest in the G2 phase^71^. Another study implicated PRODH1 in the regulation of the enzyme cyclooxygenase 2 (COX-2), whereby increased expression of PRODH1 inhibited expression of COX-2 (Fig. 3)^76^. COX-2 is an enzyme involved in prostaglandin biosynthesis and its expression correlates with worse prognosis in several malignancies^77^. Indeed, COX-2 inhibitors, such as celecoxib, have widespread anticancer activities and can induce apoptosis in cancer cells^78^. Of note, preliminary indications in oral squamous cell carcinoma suggest that celecoxib treatment increases levels of PRODH1, although it has yet to be established whether such an increase contributes to celecoxib-mediated suppression of cell growth and viability^79^. In agreement with its role as tumor suppressor gene, reduced expression of *PRODH1* has been reported in several cancers, with more consistent downregulation observed in renal cancers and cancers arising from the digestive tract^64,71,76,79–81^.

As aforementioned, the PRODH1 enzyme localizes in the mitochondrial inner membrane and transfers electrons directly to Coenzyme Q1 (CoQ1)^51,82^. In this context, the role of PRODH1 in cancer is double-faced. In conditions of nutrient limitation, PRODH1 reduces CoQ1 directly and contributes to ETC function and energy production^51,82^. However, as the levels and activity of PRODH1 increases, the consequent generation of ROS causes downregulation of several components of the ETC and compromises mitochondrial respiration^51^. Interesting as they are, these data also outline the limitations of interpreting experiments performed using non-physiological overexpression. Notwithstanding, in cancer cell lines from different tumors, PRODH1 is upregulated in response to energetic stress and nutrient deprivation triggered by hypoxia or glucose restriction^82,83^. In these challenging circumstances, cells engage the AMP-protein kinase (AMPK). AMPK is a sensor of cellular energy status, which is activated under conditions of high AMP/ATP ratio caused by stressors, including hypoxia or nutrient deprivation. Once activated, AMPK rewires cellular metabolism by stimulating catabolism to revert the energetic deficit and by triggering autophagy to counteract nutrient limitation^84^. AMPK also stimulates transcription from the *PRODH1* gene and the accumulation of PRODH1 enzyme contributes to cell survival^82,85^. Under glucose restriction, PRODH1 degrades L-proline to sustain mitochondrial activity and ATP production, whereas, in hypoxic cells, PRODH1-mediated increase in ROS levels contributes to activation of autophagy (Fig. 3)^82,85^. Therefore, the outcome of ROS production by PRODH is context-dependent: it is pro-apoptotic in normoxia but stimulates protective autophagy in hypoxia^83^. The link between hypoxia and proline metabolism goes deeper; the main outcome of the hypoxia response protein HIF1-α is to promote expression of matrix metalloproteinase and degradation of the ECM. This causes an increase in hydroxyproline availability, which has been reported to contribute to HIF1-α stabilization and cancer cell survival in hepatocellular carcinoma cells exposed to hypoxia^86^.

The ability of PRODH1 to support mitochondrial function during nutrient stress proved essential in pancreatic cancer^61^. Pancreatic tumor cells organize into glandular structures surrounded by a dense, collagen-rich fibrotic tissue that hinders vascularization and reduces oxygen and nutrient availability^87^. In this instance, tumor cells maintain a functional TCA cycle through PRODH1 activity and collagen breakdown, as carbon obtained from the catabolism of collagen-derived L-proline feeds into the TCA cycle^61^. Chemical inhibition or genetic depletion of PRODH1 in pancreatic cancer cells compromise mitochondrial oxygen consumption, which can be recovered by external L-proline supplementation, and dramatically reduces cell growth in vitro or in vivo^61^.

*PRODH1* has been shown to contribute to non-small cell lung cancer (NSCLC) progression through both cell-autonomous mechanisms and the promotion of a pro-tumorigenic inflammatory milieu^88^. Increased expression of *PRODH1* occurs in a subset of NSCLC specimens. Consistent with previous findings^67,69^, this increased expression is p53-dependent, which begs the questions whether NSCLC reliance on PRODH1 persists in cancers in which p53 is mutated^88^. Functionally, PRODH activity drives cell proliferation and tumor growth in vivo and promotes cell migration and invasion^88^. Moreover, expression of PRODH1 triggers production of intracellular ROS, which, in turn, drives the expression of pro-inflammatory cytokines^88^. Whether those cytokines play a direct role in promoting carcinogenesis necessitates further investigation. However, their increased expression in lung adenocarcinoma specimens correlates with poor prognosis^88^.

A role for PRODH in promoting breast cancer has also been reported^89^. When grown in 3D culture, breast cancer cells increase the expression of *PRODH1* and augment proline catabolism. Genetic depletion of *PRODH1* or pharmacological inhibition of PRODH1 using the reversible inhibitor L-tetrahydro-2-furoic acid (L-THFA) impair growth of breast cancer cells^89^ in vitro. However, L-THFA treatment failed to inhibit growth of primary tumors in vivo when breast cancer cells were orthotopically injected in recipient mice^89^.

Overall, these reports unambiguously demonstrate that, in some relevant circumstances, *PRODH1* supports tumor progression and is a potential anticancer drug target^89,90^. The attribution of *PRODH1* to the category of tumor suppressor is, therefore, context-dependent, emphasizing the notion that metabolic adaptations are cancer-specific.

## The unequivocal role of *PYCR1* in cancer

*PYCR1* was initially identified as a pro-tumorigenic gene in an RNA-interference screening in breast cancer^91^. More recently, a meta-analysis covering 1981 tumors from 19 different types of cancers highlighted that *PYCR1* is one of the most commonly overexpressed metabolic genes in human cancer^92^. A wider rewiring of proline metabolism in favor of proline biosynthesis was suggested by Phang’s lab. The oncogenic transcription factor c-Myc is often upregulated in cancer, and it is known to rewire tumor cell metabolism, especially stimulating glutamine catabolism to fuel the TCA cycle^93,94^. Working with prostate cancer and Burkitt lymphoma cells, Phang’s team demonstrated that c-Myc triggers downregulation of the PRODH1 enzyme through increased expression of Mir-23b*, while enhancing transcription from the *PYCR1* and *ALDH18A1* (encoding P5CS) genes^95^. Therefore, c-Myc appears to promote a switch from proline catabolism to proline anabolism in cancer, reshuffling part of the cellular glutamine toward proline biosynthesis.

Increased expression of *PYCR1* has since been confirmed in different cancers, including melanoma, NSCLC, renal cell carcinoma, breast cancer, colorectal cancer, prostate cancer, hepatocellular carcinoma, and isocitrate dehydrogenase 1 (IDH1)-mutant gliomas^41,42,64,96–102^. In some instances, *PYCR1* expression is a reliable diagnostic biomarker which predicts poor prognosis in patients with melanoma^96^, breast cancer^64,101^, renal cell cancers^100,103^, and NSCLC^98^. Studies that employed genetic depletion of *PYCR1* have confirmed a functional role for *PYCR1* in promoting tumor progression and cancer cell survival in colorectal cancer cells^104^, NSCLC cells^42,97,98^, renal cancer cells^64,103^, prostate cancer cells^105^, and IDH1-mutant gliomas^106^.

Different mechanisms have been proposed to explain the ability of PYCR1 to support tumor progression, which could suggest a remarkable degree of pleiotropy and tissue specificity.

## Proline biosynthesis and protein translation

Intuitively, PYCR1 activity might satisfy cancer cells’ increased demand for the NEAA L-proline. Indeed, in some experimental conditions, external supplementation of L-proline partially rescues the reduced cellular proliferation and survival caused by perturbations of the proline biosynthesis pathway^42,64,107^. A subset of clear cell renal cell carcinomas and the majority of invasive ductal breast carcinomas show increased expression of *PYCR1* mRNA^64,101^. In these cancers, PYCR1 activity provides L-proline necessary for protein biosynthesis. Cancer cells depleted of PYCR1 display impaired progression in growth-limiting conditions, such as in vivo in recipient mice or in the presence of low glutamine in vitro^64^. This growth defect is associated with ribosome stalling at proline codons because of reduced aminoacylation of the corresponding tRNAs. Consistent with a direct role for L-proline production, external supplementation of L-proline is sufficient to rescue the growth defect^64^. That L-proline availability might be limiting to cancer cells is in agreement with the reduced fasting plasma levels of L-proline in patients with lymphoma and some sarcomas, a reduction that is not correlated to weight loss or circulating levels of other amino acids^108^.

Modulation of protein translation by PYCR1 activity has been reported, where disruption of proline metabolism affects the two main pathways that regulate protein synthesis: the PI3K/Akt/mTOR pathway and the amino acid response (AAR) pathway (Fig. 3). The mammalian (or mechanistic) target of rapamycin (mTOR) kinase is a major regulator of organismal response to nutrient availability. When nutrients are abundant and in response to receptor tyrosine kinase activation of the PI3K/Akt pathway, the mTOR complex 1 (mTORC1), which, together with the mTOR kinase and other components, includes the essential scaffolding protein regulatory-associated protein of mTOR (RAPTOR), stimulates protein translation through direct phosphorylation of downstream targets EF4B and S6K kinase^109,110^. In melanoma cells, knockdown of PYCR1 causes decreased activity of the Akt pathway and reduces the expression of RAPTOR, suggesting a downstream inhibition of protein synthesis^96^. Similarly, RNA-interference suppression of PYCR1 decreases levels of activated phospho-Akt and activated phospho-mTOR in renal cancer cells^103^.

The AAR pathway is engaged in response to amino acid starvation. The general control nonderepressible 2 (GCN2) kinase is an amino acid sensor activated by uncharged tRNAs that accumulate in response to low amino acid availability. Once activated, GCN2 inhibits translation through phosphorylation of the eukaryotic initiation factor eIF2 and, concomitantly, stimulates the Cap-independent translation of the transcription factor activating transcription factor 4 (ATF4)^111,112^. ATF4 drives a transcriptional program that enhances amino acid metabolism, including increasing the transcription of the proline metabolism genes *ALDH18A1* and *PYCR1*^113,114^. Compared to normal tissue controls and non-transformed melanocytes, melanoma cells express higher levels of proline biosynthesis enzymes and display higher intracellular levels of L-proline^41,107^. Knockdown of P5CS reverts the increase in intracellular L-proline and, concomitantly, compromises cellular proliferation and viability, and severely reduces protein synthesis^107^. Notably, those effects depend on activation of the AAR pathway and are rescued by supplementation of the growth media with L-proline, which partially restores protein translation and cellular viability, preventing the activation of the AAR pathway^107^. Whether proline synthesis is associated with regulation of the mTOR pathway in melanoma cells is controversial^107,115^.

A remarkable example of direct reliance on L-proline production has been recently reported in lung adenocarcinoma. Guo and colleagues identified a cross talk between the ECM and proline metabolism mediated by the protein kindlin 2^42^. Kindlin 2 is a ubiquitously expressed protein that localizes to focal adhesions to regulate integrin-mediated cell–ECM adhesion^116,117^. The authors show that a subset of kindlin 2 localizes to the mitochondria where it binds to PYCR1 (and PYCR2). Remarkably, this interaction stabilizes PYCR1 protein and increases proline biosynthesis. In addition, kindlin 2 localization to the mitochondria is stimulated by ECM stiffness: when cells are plated on a stiff ECM substrate more kindlin 2 is found in the mitochondria, bound to PYCR1. Consequently, the rate of proline biosynthesis responds to changes in ECM stiffness with functional implication for tumorigenesis. Lung adenocarcinoma cells where kindlin 2 is knocked-out using CRISPR/Cas9, together with impaired proline metabolism, display reduced proliferation and survival. Overexpression of PYCR1 or external supplementation of L-proline is sufficient to restore proliferative competence in kindlin 2-null cells^42^. Unfortunately, the authors did not investigate whether the primary role of L-proline was to sustain protein translation, and the function of L-proline biosynthesis in this context remains to be fully elucidated.

## Proline biosynthesis and the TCA cycle

Similar to what has been observed for PRODH, the link between proline biosynthesis and the metabolism of glutamine and NAD(P)H necessarily affects mitochondrial function. This is the case for a subset of human gliomas characterized by mutations in the *IDH1* gene, which codes for the NADP-dependent cytosolic IDH1 enzyme. Cancer-related mutations in *IDH1* are heterozygous missense alterations most commonly generating an arginine to histidine substitution in residue 132 (R132H)^118^. This mutation has been shown to change the enzymatic activity of the IDH1 enzyme; IDH1 oxidation of isocitrate to α-ketoglutarate is replaced by NADPH-coupled reduction of α-ketoglutarate to (R)-2-hydroxyglutarate^119^. 2-Hydroxyglutarate is known as an oncometabolite due to its direct role in promoting tumor progression through inhibition of α-ketoglutarate-dependent dioxygenases, such as the TET-family of DNA demethylases and the HIF1-α prolyl hydroxylases, thereby altering the epigenetic landscape of tumor cells and the response to hypoxia^120,121^. IDH1-mutant gliomas express higher levels of PYCR1 than their wild-type counterparts and engage proline biosynthesis from glutamate^106^. In this case, the ability of PYCR1 to drive cancer progression does not rely on the end product L-proline, as *IDH1*-mutant glioma cells release most of the newly synthesized L-proline extracellularly^106^. Rather, it is the parametabolic regulation of NADH metabolism that confers on PYCR1 the ability to sustain TCA cycle activity. PYCR1 oxidation of NADH to NAD^+^ allows the TCA cycle to progress independently of oxygen consumption^106^. This activity might be essential in tumors, such as gliomas, that experience hypoxia and therefore limited ETC activity. Conditions that compromise mitochondrial ETC force cells to increase flux through the glutamine-proline pathway, in order to dissipate electron buildup through NADH oxidation by P5CS and PYCR enzymes^122,123^. This property of PYCR1 to act as a “mitochondrial vent” has wider implications. Recent findings demonstrate that, in fibroblasts, TGF-β signaling enhances L-proline production from glutamate through SMAD4-dependent transcription of L-proline metabolism genes, including *ADH18A1*, *PYCR1*, and *PYCR2*^124^. TGF-β signaling increases the synthesis of collagen and ECM deposition. It also enhances mitochondrial redox potential by channeling glucose and glutamine for oxidation via the TCA cycle, in order to increase energetic output. This leads to TCA cycle activity exceeding the capacity of the ETC to convert mitochondrial redox potential into ATP. In this circumstance, proline biosynthesis supports collagen production and alleviates mitochondrial hyperpolarization through oxidation of NADH and diversion of glutamine-derived carbon away from the TCA cycle^124^.

## Additional pathways modulated by *PYCR1*

In the context of different cancers and experimental models, researchers have identified different signaling pathways that are influenced by proline biosynthesis (Fig. 3). In colorectal cancer cells, *PYCR1* knockdown is associated with reduced activity of the MAPK pathway and NF-kB signaling, phenotypes that seem to depend on signal transducer and activator of transcription (STAT) 3-mediated signaling^104^. Indeed, Yan and colleagues found that ectopic expression of the transcription factor STAT3 reverses the proliferative reduction observed following *PYCR1* knockdown^104^. According to their data, PYCR1 binds directly to STAT3 to promote its transcriptional activity. A role for PYCR1 in regulating the MAPK p38 was confirmed in NSCLC, where p38 MAPK activation depends on PYCR1 expression and contributes to PYCR1-mediated increase in cell survival and proliferation^98,125^. In hepatocellular cancer cells, shRNA-mediated knockdown of PYCR1 in vitro resulted in significantly decreased activation of the stress-activated protein kinase/c-Jun NH(2)-terminal kinase (SAPK/JNK) signaling pathway and the insulin receptor substrate 1^102^. However, the implications of these changes for tumorigenesis are unclear.

## Other proline enzymes

Whilst it is generally acknowledged that *PYCR1* plays a major role in cancer progression, some preliminary data suggest a pro-tumorigenic role for the *PYCR2* gene as well. Confirming the high dependence of melanoma on proline metabolism, siRNA-mediated knockdown of *PYCR2* was found to reduce proliferation and provoke a mild increase in apoptosis in melanoma cells^115^. Depletion of PYCR2 also caused activation of AMPK kinase. In line with AMPK ability to inhibit the mTOR pathway^84^, mTOR activation was impaired in PYCR2-knockdown cells with a concomitant increase in autophagy markers^115^. These data are notable as they unveil the regulation of the mTOR pathway by another PYCR isoform. However, the regulation of mTOR by proline biosynthesis enzymes in melanoma has not been consistently observed^107^.

Melanoma cells have increased PC5CS protein levels compared to melanocyte controls and, as previously reported, P5CS is essential to sustain protein translation and proliferation^41,107^. In Burkitt lymphoma and prostate cancer cell lines the oncogene c-Myc causes a significant increase in P5CS expression^95^. Other findings have reported that the expression of the P5CS gene *ALDH18A1* is increased and contributes toward enhanced L-proline biosynthesis in fibroblasts stimulated with TGF-β and in hepatocellular carcinoma cells subject to hypoxic environment^86,124^. In the DLD-1 colorectal cell line, induction of p53 resulted in increased expression of the long isoform of P5CS^38^. However, whether this is functionally meaningful is doubtful, as ectopic expression of both the short and long P5CS isoforms in different cell lines had no overt effect on their proliferation or survival^38^. The role of P5CS is likely to be context-specific, as, in breast cancer, CRISPR/Cas9-mediated knockout of P5CS had no impact on tumor growth, but it sensitized cancer cells to pharmacological inhibition of lipogenesis, indicating that inhibition of proline biosynthesis could synergize with therapies targeting lipid metabolism^122^. Recently, increased expression of OAT has been shown to correlate with NSCLC progression, and OAT activity supports proliferation and metastatic spread of NSCLC cells^126^.

In addition to *PRODH1*, p53 also induces the expression of *ALDH4*, which codes for P5CDH^127^. Therefore, the p53 transcriptional program stimulates proline catabolism to glutamate. However, whereas PRODH expression has been linked to p53-induced apoptosis, induction of P5CDH has a protective role from oxidative stress. RNA-interference-mediated knockdown of P5CDH sensitizes HCT-116 colorectal cancer cell lines to p53-induced cell death, whereas, H1299 lung cancer cells that overexpress P5CDH showed significantly lower intracellular ROS levels than parental cells when challenged with hydrogen peroxide or UV^127^. In addition, analysis of RNA-seq data unmasked an exon skip (a common pattern of alternative splicing) in the *P5CDH* gene that predicts poorer survival in rectal cancer patients^128^, although the physiological implications of this alternative splicing event are unknown. Overall, the contribution of *P5CDH* to tumor progression is likely to be limited, as *P5CDH* has not been associated with progression of breast cancer and NSCLC^89,128–131^.

PYCR3 has been so far poorly investigated and any link to tumorigenesis remains unexplored. However, a recent association between PYCR3 and cells’ adaptation to L-proline starvation has been reported. Sahu and colleagues identified subsets of cancer cells that rely on external supplementation of L-proline for proliferation and survival and undergo robust ER stress if starved of L-proline. These cells express lower levels of both *ALDH18A1* and *PYCR3* gene and RNA-interference-mediated knockdown of *PYCR3* reduced colony formation in L-proline starved cells. The authors conclude that increased L-proline biosynthesis through the P5CS-PYCR3 axis protects cells from ER stress and contributes to proliferation and survival in L-proline limiting conditions^132^. Increased expression of *PYCR3* has also been identified in a pan-cancer systematic analysis of metabolic adaptations in response to hypoxic environment, again confirming the critical role of proline metabolism in response to oxygen limitation^123^. These findings indicate a potential role for PYCR3 in tumorigenesis that might be dependent on tumor cell adaptation to L-proline availability and the hypoxic tumor microenvironment.

## Proline and ROS

The increase in intracellular L-proline in response to stress has been known for many years in plants, where it provides protection against oxidative stress^133^. Indeed, carbon atoms within the L-proline ring can efficiently react with and quench some species of radicals^134^. Cancer cells face the challenge of coping with oxidative stress and have developed several adaptive mechanisms to manage oxidative damage^5^. Notably, L-proline has been shown to mitigate photo-oxidative damage caused by exposure to UV light^135^, and supplementation of L-proline in the culture medium protects human cells from hydrogen-peroxide-induced cell death^136^. Importantly, this protection is substantial at a nearly physiological concentration of the amino acid (0.5 mM in culture media vs 0.2–0.3 mM normally found in the human serum)^136^. The role of L-proline in balancing cellular oxidative burden in cancer needs to be confirmed in physiologically relevant models. Alternatively, proline metabolism regulates ROS production directly in the mitochondria. As previously mentioned, PRODH is known to increase intracellular ROS^69^. In contrast, active proline biosynthesis activity has been shown to diminish ROS production, probably by easing the hyperpolarization of mitochondrial triggered by accumulation of reduced NADH or intense TCA cycle activity^106,124^. Emphasizing the anti-oxidative function of proline biosynthesis enzymes, Kuo and colleagues showed that PYCR1 and PYCR2 bind to the ribonucleotide reductase small subunit RRM2B and support its anti-oxidant activity^40^. RRM2B, also known as the p53 inducible p53R2, has been shown to protect cells from oxidative damage^137,138^. Kuo and colleagues found that PYCR1 and PYCR2 directly interact with RRMB2 in the mitochondria and confer resistance to hydrogen-peroxide-mediated cell death stimulating the anti-oxidant activity of RRM2B^40^. Intriguingly, together with the data on melanoma^115^, this work provides an additional indication that PYCR2 might play a significant role in carcinogenesis. PYCR1 has also been linked to protection from ROS in neuronal cells via direct binding to the protein DJ-1^139^. Beyond its known role in neuronal physiology and Parkinson’s disease, DJ-1 is overexpressed in several cancers and has been linked to worse prognosis^140–142^. Although any interaction between PYCR1 and DJ-1 in cancer is yet to be established, these data warrant further investigation into their possible cooperation in promoting tumorigenesis by conferring protection to oxidative stress.

However, exceptions to the anti-oxidant function of PYCR1 have been reported. Kuo and colleagues unveiled an interaction between PYCR1 and the mitochondrial chaperone protein Lon^125^. Lon expression is induced in response to stress, such as the unfolded protein response, hypoxia, and oxidative stress, to safeguard cells’ ability to proliferate and survive^143–146^. In cancer cells, Lon expression triggers mitochondrial ROS production and, surprisingly, this depends on Lon interaction with PYCR1. The authors showed that Lon expression induces accumulation of PYCR1 and that the two proteins bind to each other and cooperate in enhancing ROS generation^125^. Of note, overexpression of PYCR1 itself in cancer cells from different tumors is associated with increased intracellular ROS levels. The Lon/PYCR1-mediated induction of ROS has notable downstream effects, as it triggers ROS-dependent activation of the p38 MAPK and NF-κB pathways to promote an inflammatory milieu, which can contribute to tumorigenesis^125^. Similarly, in a subset of lung cancers that overexpress PRODH, tumor progression is driven by PRODH-mediated increase in ROS, which acts as signaling molecules stimulating the expression of pro-inflammatory cytokines^88^. In this instance, PRODH-mediated generation of ROS is blunted upon knockdown of proline biosynthesis enzymes PYCR1, PYCR2, and PYCR3^88^. This suggests that by recycling P5C back to L-proline, PYCR enzymes fuel PRODH activity and, indirectly, act as pro-oxidants^88^. Indirect evidence in support of this possibility is the reported anti-oxidant effect of P5CDH expression triggered in response to p53 activation^127^. Indeed, by metabolizing P5C to glutamate, P5CDH could compete with PYCRs for substrate utilization, reducing L-proline availability for oxidation and ROS generation by PRODH.

These findings are puzzling to the extent that they contradict previous robust indications that PYCR1 activity would rather dampen cellular ROS. It will be necessary to establish whether the net effect of PYCR1 on mitochondrial ROS production is context-dependent and whether it depends on the concomitant expression of PRODH. Of course, it cannot be ruled out that yet-to-be identified mechanisms regulating PYCR1 interaction with mitochondrial metabolism are responsible for these conflicting outcomes.

## The proline cycle in cancer

Hagedorn and Phang first suggested the existence of a proline cycle over 30 years ago^147,148^. In the cycle, PRODH oxidizes L-proline to P5C in the mitochondria, a reaction coupled with the transfer of electrons to the mitochondrial ETC. P5C is then reduced back to L-proline through the action of the PYCR enzymes in the cytosol, a reaction coupled with the oxidation of NADPH. The outcome of this cycle is to transfer electrons from the cytosol to the mitochondria, similarly to what observed with traditional mitochondrial shuttles, such as the malate–aspartate shuttle or the glycerol–phosphate shuttle (Fig. 4). However, the proline cycle entails another important metabolic consequence. Since proline biosynthesis in the cytosol is mostly dependent on NADPH oxidation, a sustained activity of the cycle will result in a higher NADP^+^/NADPH ratio^60^. As initially indicated by Krebs, the NADP^+^/NADPH ratio is the rate-limiting variable that controls flux through the PPP^149^, and an increase in NADP^+^ levels will stimulate the oxidative arm of the PPP leading to increase ribose and, consequently, nucleotide biosynthesis. Evidence exists that proline metabolism might accelerate the rate of PPP and the metabolic flux of nucleotide biosynthesis, fostering support to the proline cycle model^81,82,150^. Moreover, the aforementioned pro-tumorigenic role of PRODH in breast and lung cancers necessitates the concomitant activity of the PYCR enzymes, indicating that P5C recycling is important for sustaining PRODH activity^88,89^. These findings can be partially reconciled with the existence of a proline cycle, but an issue with the pathway compartmentalization emerges. The initial proline cycle model suggested a series of compartmentalized reactions of oxidation of L-proline in the mitochondria and reduction of P5C in the cytosol. This would suggest that the main PYCR enzyme involved should be the elusive cytosolic PYCR3, which also has the highest affinity for NADPH^41^. However, there is no sound evidence implicating PYCR3 in cancer or the PPP. Moreover, this would not explain why PRODH pro-tumorigenic properties rely on the expression of mitochondrial PYCR1 and PYCR2. The presence of PYCR1 in the cytosol, at least in minimal quantity, has been reported, as well as its potential localization on the outer mitochondrial membrane^42,43^. If confirmed, these possibilities could help reconcile the proline cycle model with our understanding of the compartmentalization of proline metabolism. Otherwise, at least in some circumstances, cancer cell proliferation might be sustained not by the proline cycle, but by recycling of P5C from L-proline, an alternative option that would weaken links to the PPP.

## Emerging pathways linked to proline metabolism

### Invasion and metastasis

PRODH expression has been reported to drive metastatic spread from breast cancer^89^. In clinical specimens, *PRODH1* expression is significantly higher in metastatic versus non-metastatic breast cancers. This is reflected in the reduced content of proline in lung metastasis isolated from mice orthotopically implanted with the mouse breast cancer cells 4T1^89^. Pharmacological inhibition of PRODH in vivo using L-THFA results in reduced metastatic spread to the lung^89^.

Tumor spread is thought to rely on the epithelial–mesenchymal transition (EMT). During EMT, epithelial tumor cells undergo a molecular reprogramming that causes the loss of epithelia adhesion molecules and the acquisition of a fibroblast-like morphology with increased ability to migrate and invade^151^. Together with its role in metastatic spread in breast cancer, PRODH has been shown to instigate EMT reprogramming and to increase migration and invasion of NSCLC cells^88^. Although not formally proved, the cross talk between proline catabolism and biosynthesis is likely to play a role also in regulation of invasiveness. Indeed, *PYCR1* expression is increased in metastatic breast cancer. Its genetic depletion in breast, lung cancer, renal carcinoma cells, and melanoma reduces cells’ ability to migrate and invade in in vitro essays^96,99,101,103^. In addition, supplementing L-proline in the culture medium stimulates proliferation and induces a mesenchymal invasive phenotype in mouse embryonic stem cells (mESCs)^152,153^. mESCs experience an intrinsic L-proline starvation in traditional culture medium and activate the AAR pathway. Notably, L-proline is the only NEAA able to alleviate the AAR, a step necessary to induce EMT-like features^153^. Additional data indicate that L-proline supports protein synthesis and induces reprogramming of the cellular histone methylation profiles in mESCs, therefore reshaping the transcriptomic output^152,153^. It will be interesting to investigate whether these mechanisms persist in cancer cells, especially in the context of IDH-mutant tumors, where tumor-related epigenetic changes are induced by the oncometabolite 2-hydroxyglutarate^120,121^.

### Cancer stem cells (CSCs)

Tumors are thought to arise from or to be driven by a subset of cells that exhibit properties of stem cells. CSCs possess the ability to self-renew, are highly tumorigenic and, being resistant to chemotherapy, are believed to be responsible for the persistence and relapse of cancer^154^. Recently, Sharif and colleagues reported an unexpected role for the p53 homolog TAp73 in safeguarding CSCs^155^. The transcription factor TAp73 was initially shown to possess tumor suppressor functions^156,157^. However, other findings implicated TAp73 in the regulation of cellular metabolism, showing that it promotes pro-proliferative anabolism, mitochondrial respiration, and metabolic adaptation in response to oxidative stress^158–163^. Sharif and colleagues demonstrated that depletion of TAp73 in CSCs reduces their survival and ability to form tumors in vivo^155^. This surprising phenotype is associated with a rewiring of amino acid metabolism: knockdown of TAp73 decreases the expression of *PYCR1* and *GLS* (coding the glutamine metabolizing enzyme glutaminase) while increasing *OAT* expression^155^. The final outcome is a reshuffling of proline metabolism toward the urea cycle. The effective relevance of proline metabolism in maintaining CSCs survival remains to be elucidated, since external supplementation of L-proline is ineffective in restoring CSCs fitness following TAp73 depletion^155^. However, TAP73 is necessary to maintain redox balance and to regulate autophagy through the AMPK-mTOR axis in CSCs^155^, all pathways linked to proline metabolism. These data on CSCs could also be reconciled with the role of proline metabolism in EMT and invasion, since during the process of EMT cancer cells acquire features of stem cells^164^.

### The tumor microenvironment (TME)

Rich in ECM and collagen the TME is an important reservoir of L-proline for cancer cells^82,89^. However, proline metabolism can also influence non-tumor cells within the stroma. Indeed, the transcription factor c-Myc triggers expression of *PYCR1* and *ALDH18A1* in activated T lymphocytes, and there is evidence that proline metabolism contributes to the reprogramming of tumor-infiltrating macrophages^125,165,166^. Two major molecular subtypes of macrophages populate the TME; M1 macrophages are very effective antigen-presenting cells and support anti-tumor immune response, whereas the alternative M2 phenotype exhibits anti-inflammatory and pro-tumorigenic activity. M2 cells influence multiple features of tumor development, including cell survival, proliferation, stemness, and invasiveness along with angiogenesis and immune evasion^167^. M2 polarized macrophages display an intense rewiring of NEAA metabolism, including arginine–glutamine–proline metabolism^165^. As previously discussed, the mitochondrial chaperon Lon interacts with PYCR1 in cancer cells to increase intracellular ROS production and activate a p38-NF-kB signaling axis^125^. The latter, in turn, stimulates cancer cells to secrete cytokines, including TGF-β, IL-13, IL-6, and VEGF-A, which trigger M2 macrophage polarization^125^. A role for PRODH in supporting a pro-inflammatory milieu has been reported also in NSCLC^88^. Notably, the metabolite P5C could also act as a signaling molecule between tumor cells and cells of the immune system. Prostate tumors release P5C to inhibit the proliferation of T lymphocytes and cytokine production, hence suppressing the immune response^168^. These data suggest that proline metabolism can have a major impact on shaping the tumor inflammatory milieu and promoting immune evasion.

## Concluding remarks and future perspectives

The evidence provided here outlines a major, and perhaps so far under-appreciated, role for proline metabolism in influencing the process of carcinogenesis. Proline metabolic enzymes work downstream of p53 to promote cell death and tumor suppression, but, in other contexts, they behave as powerful oncogenic proteins in support of tumor growth and metastatic spread. Notwithstanding its relevance to tumorigenesis, on this day there are no clinically relevant pharmacological compounds to target the proline pathway.

The amount of literature on the subject has accumulated rapidly in the past few years; this has amplified the amount of information available, but not necessarily its clarity. Moving forward, it will be pivotal to develop appropriate tools to investigate the role of proline metabolism in cancer. The use of RNA-interference-based approaches to gene depletion has been informative, but mostly limited to cell culture work and potentially affected by the lack of selectivity when targeting conserved isoforms. The engineering of more physiologically relevant models, such as inducible knockout mouse models or CRISPR/Cas9 modified organoids, will be key to clarify the function of the different enzymes and their isoforms in vivo, as elegantly demonstrated by the generation by Liu and colleagues of a CRE-inducible P5CS knockout mouse model for the analysis of breast cancer tumorigenesis^122^. These genetically neat models could unravel the different functions of PYCR isoforms in cancer and finally shed light on the role of the neglected *PYCR3* gene in physiology and disease, not to mention its potential contribution to the proline cycle. More generally, it will be key to address in which tumors and in which context the metabolism of proline is preferentially unidirectional, and which malignancies rely on the coexistence of PYCR and PRODH activities. In this regard, large cancer datasets should be mined to address expression of proline metabolism genes comprehensibly, rather than limiting the analysis to the expression of single isoforms. This will enable a stratification of tumors based on gene expression profiles of the whole pathway. Flux metabolomics should be extensively employed to aid discovery and inform about the cross talk between proline metabolism and the wider cellular metabolic network. These investigations will aid efforts to target proline metabolism pharmacologically and could inform on potential toxicities. Indeed, although preliminary evidence indicates that in vivo inhibition of PRODH is achievable^89^, mutations in proline metabolism genes have been implicated in metabolic syndromes known as prolinemia and impaired development of the nervous system, the epidermis and other organs^169–176^.

Efforts should also be dedicated to unmasking the interaction between proline metabolism and chemotherapy. Preliminary data suggest that inhibiting proline biosynthesis enhances sensitivity of colorectal cancer cells and hepatocellular carcinoma cells to standard of care chemotherapy agents 5-FU and sorafenib, respectively^86,104^. On the other hand, PRODH has been implicated in cell death induced by p53-inducing DNA-damaging drugs^69^ and to contribute to anti-tumor activity of celecoxib^79^ and troglitazone, a peroxisome proliferator-activated receptor γ (PPARγ) agonist that induces apoptosis in several cancers^177^. This information will help prioritize cancers for mechanistic studies and preclinical assessments of new compounds targeting proline enzymes.

Post-translational modifications of proline metabolic enzymes deserve a final mention as a research area likely to receive growing attention in the future. In their recent study, Chen and colleagues reported that PYCR1 activity is regulated through acetylation of lysine K228, located in the C-terminal dimerization domain. The CREB-binding protein (CBP) acetylation of K228 is counteracted by the activity of the NAD^+^-dependent, mitochondrial SIRT3 deacetylase. Importantly, acetylation impairs the ability of PYCR1 to polymerize and partially reduces its enzymatic activity, and impacts cellular proliferation^178^. Of note, the regulation of PYCR1 acetylation is dictated by a NAD^+^-dependent enzyme, suggesting that PYCR1 enzymatic output is wired to the NAD^+^/NADH balance. More generally, here we proffer the contention that, whilst the post-translation modifications of proline metabolizing enzymes are largely unknown, they very likely play major roles in the regulation of this metabolic pathway in cancer. How they influence proline metabolism during tumorigenesis warrants further investigation.
